# Gray matter volume covariance networks are associated with altered emotional processing in bipolar disorder: a source-based morphometry study

**DOI:** 10.1007/s11682-021-00541-5

**Published:** 2021-09-21

**Authors:** Alessandro Miola, Nicolò Trevisan, Arcangelo Merola, Francesco Folena Comini, Daniele Olivo, Matteo Minerva, Silvia Valeggia, Tommaso Toffanin, Angela Favaro, Renzo Manara, Fabio Sambataro

**Affiliations:** 1 Department of Neuroscience (DNS), University of Padova, Via Giustiniani 5, Padova, Italy; 2 Padova Neuroscience Center, University of Padova, Padova, Italy

**Keywords:** Bipolar disorder, Voxel-based morphometry, Source-based morphometry, Cognition, Emotional processing

## Abstract

Widespread regional gray matter volume (GMV) alterations have been reported in bipolar disorder (BD). Structural networks, which are thought to better reflect the complex multivariate organization of the brain, and their clinical and psychological function have not been investigated yet in BD. 24 patients with BD type-I (BD-I), and 30 with BD type-II (BD-II), and 45 controls underwent MRI scan. Voxel-based morphometry and source-based morphometry (SBM) were performed to extract structural covariation patterns of GMV. SBM components associated with morphometric differences were compared among diagnoses. Executive function and emotional processing correlated with morphometric characteristics. Compared to controls, BD-I showed reduced GMV in the temporo-insular-parieto-occipital cortex and in the culmen. An SBM component spanning the prefrontal-temporal-occipital network exhibited significantly lower GMV in BD-I compared to controls, but not between the other groups. The structural network covariance in BD-I was associated with the number of previous manic episodes and with worse executive performance. Compared to BD-II, BD-I showed a loss of GMV in the temporal-occipital regions, and this was correlated with impaired emotional processing. Altered prefrontal-temporal-occipital network structure could reflect a neural signature associated with visuospatial processing and problem-solving impairments as well as emotional processing and illness severity in BD-I.

**Supplementary Information:**

The online version contains supplementary material available at 10.1007/s11682-021-00541-5.

## Introduction

Bipolar disorders (BD) affect up to 4% of the general population (Merikangas et al., 2007). These disorders are often unrecognized due to the heterogeneity of the clinical features (Hirschfeld et al., 2003), which results in delayed diagnosis, inadequate treatment, high medical costs, and disability, partly due to cognitive alterations (Keck et al., 2008). Bipolar type-I (BD-I) and type-II (BD-II) are the major subtypes of BD: BD-I presents with more severe and long-lasting mood symptoms along with psychosis and greater functioning deficits; BD-II has a more chronic course with frequent depressive and shorter euthymic phases (Baldessarini et al., 2000). In BD, cognitive impairment has been reported in several domains, including attention, processing speed, executive functions, and memory (Bora & Özerdem, 2017) as well as in hot cognition, (Işık Ulusoy et al., 2020). Cognitive deficits are stable and relatively independent of mood changes and can be detected in 40–60% of euthymic patients (Srivastava et al., 2019). BD-I has more severe and global impairment when compared with BD-II, albeit negative results have been reported (Bora, 2018).

Neuroimaging literature has reported anatomical alterations in several regions. Previous meta-analyses of voxel-based morphometry (VBM) studies in BD indicated reduced gray matter volume (GMV) in the frontal-insular cortex and temporal regions (Bora et al., 2012). A further meta-analysis of 50 studies, revealed reduced GMV in the prefrontal cortex, temporal cortex, insula, and anterior cingulate cortex in BD (Wang et al., 2019). Few small studies have investigated BD subtype differences showing shared GMV alterations that are less pronounced in BD-II (Maller et al., 2014; Abè et al. 2016). However, a large multisite study found no differences in cortical thickness and surface area between BD subtypes (Hibar et al., 2018).

Neuroimaging approaches have focused on individual regional differences, although the brain is organized in distinct networks. Source-based morphometry (SBM), a multivariate method based on structural covariance, has been proposed for detecting structural network changes (Xu et al., 2009). SBM can identify patterns of common variation among subjects, combining information across voxels to identify co-varying 'networks' with great sensitivity to identify artifacts, and has been used to study several neuropsychiatric disorders [see (Gupta et al., 2019) for a review].

In this study, our objective was to identify differences in structural network covariance in BD, as well as in distinct subtypes, using SBM. Finally, we tested the association between structural alterations and hot and cold cognition. Given the previous literature showing a smaller GMV in BD (Wang et al., 2019) along with reduced structural covariance in psychiatric disorders associated with emotional and cognitive impairment (Gupta et al., 2019), we hypothesized that patients with BD would show decreased structural network covariance, with the magnitude of this effect being the greatest in BD-I.

## Methods

### Participants

Fifty-seven patients with BD and 45 healthy controls (HC) matched with age, sex, handedness, and IQ participated in this study. Patients were diagnosed with BD using the Structured Clinical Interview for DSM-5-Patient Edition and had stable drug treatment (≥ 1 month). Exclusion criteria for all participants included age > 65 or < 18 years, history of alcohol or drug abuse in the previous six months, lifetime drug dependence, traumatic head injury with loss of consciousness, past or present major medical illness, neurological disorders, and mental retardation. In addition, controls with a history or current diagnosis of psychiatric disorders or drug treatment were excluded. The final sample included 24 BD-I and 30 BD-II. Affective symptoms were assessed using the Montgomery and Asberg Depression Rating Scale (Montgomery & Asberg, 1979), Hamilton Rating Scale for Depression (Hamilton, 1960), and the Hamilton Rating Scale for Anxiety (Hamilton, 1959), and the Young Mania Rating Scale (Young et al., 1978). Psychotic symptoms were evaluated using the Positive and Negative Syndrome Scale (Kay et al., 1987). A detailed history of mood disorders was collected and included age of onset, time of the last mood episode, number of lifetime episodes of depressive, manic, mixed, and hypomanic, and past psychotic symptoms. Family history of BD and pharmacological treatments were also recorded. This study was approved by the local Ethics Committee and all participants gave their written informed consent to participate in the study after receiving a complete explanation of the procedures.

### Neuropsychology

Neuropsychological measures were administered and included the Tower of Hanoi (TOH) for executive functions and the Facial Emotion Recognition (FER) task for emotional processing. All computerized tests were conducted using PEBL software (http://pebl.sourceforge.net/).

### Image acquisition

High-resolution structural data were acquired using a 3 T MR scanner (Philips Ingenia, Best, The Netherland) with a 32-channel quadrature head-coil using the whole-brain 3D-T1 magnetization prepared rapid gradient echo sequence in the sagittal plane (TR/TE = 6676 ms/3 ms; FOV = 240 mm; flip-angle = 8°, resolution = 1.0 × 1.0 × 1.0 mm^3^). All scans were evaluated by an expert neuroradiologist (RM) to exclude brain abnormalities.

### Imaging Processing

Data were preprocessed using the Computational Anatomy Toolbox for SPM (CAT12). Spatially normalized and segmented images were modulated and smoothed using an 8-mm full width half-maximum Gaussian kernel. First, a voxel-wise general linear model with total intracranial volume (TIV) and age as covariates was used to compare GMV across BD-I, BD-II, and HC using pairwise univariate linear contrasts. Nonparametric testing using the threshold-free cluster enhancement method with 5000 permutations was applied to family-wise correct at the cluster-level with α = 0.05.

### Source-based morphometry

Methodological details on SBM can be found elsewhere (Xu et al., 2009). Briefly, a spatial independent component analysis (ICA) was computed on all subjects’ preprocessed GMV images with the Infomax algorithm using the “Group ICA for fMRI Toolbox” (GIFT; https://trendscenter.org/software/gift/). The ‘minimum description length’ criteria were used to estimate the number of components. All GMV images were arrayed into one 99-row subject-by-image matrix. This matrix was then decomposed into a source matrix, representing the relationship between components and brain voxels (i.e., the spatial maps of the GMV components), and one mixing matrix, which indicates the relationship between participants and components. Subsequent comparisons between groups were made using mixing matrix indices, i.e., loading parameters that represent each individual’s contribution to the GMV components of the total sample. 50 bootstrapped and permuted ICA estimations were performed using the ICASSO algorithm and reliable components (coefficient of stability > 0.8) were included for further analysis. Components that were spatially correlated (r > 0.1, p < 0.001) with clusters showing GMV changes in patients with BD were retained for group analyses. First, we calculated component-related differences in GMV between BD-I, BD-II, and controls using ANCOVAs with age and TIV as covariates in the columns of the mixing matrices of the selected components, followed by Tukey's post hoc test (p < 0.05). For the visualization of the GMV component, the source matrix was re-shaped to a three-dimensional image (i.e., the same dimension as the input images), scaled to unit standard deviations (Z-maps), and thresholded at Z > 3.5. Maps of components exhibiting significant differences between the groups were then overlaid onto a normalized Montreal Neurological Institute anatomical template. Finally, we performed supplementary analyses using gender as a covariate, as well as limiting the sample to euthymic patients at the time of scan to exclude their confounding effect, if any (see Supplementary Materials for details).

### Statistical analyses

Data were compared across diagnostic groups using chi‐square tests for categorical variables and one‐way-ANOVA for continuous variables, with pairwise chi-square/Tukey post-hoc comparisons in case of statistical significance. To investigate relationship between structure, clinical and cognitive domains, we performed a Pearson’s or Spearman’s correlation between clinical data, cold and hot cognition measures, and structural changes. The level of significance was set to p < 0.05.

## Results

### Demographic and clinical characteristics (Table 1)

Detailed demographic and clinical data are presented in Table 1. BD did not differ from controls with respect to age, gender, handedness, and IQ scores (all p’s > 0.1). BD-I had a significantly greater occurrence of past psychotic symptoms (p < 0.001), lower Global Assessment of Functioning scores (p = 0.003), and drug treatment differences with lower use of antidepressants (p = 0.001), greater use of antipsychotics (p = 0.033), and a trend for increased lifetime lithium exposure (p = 0.084) relative to BD-II. BD-subtypes did not differ in terms of the illness duration, proportion of patients in the euthymic state, plasma levels of lithium, affective symptoms (all p’s > 0.1).

**Table 1:** Sociodemographic and clinical characteristics of the samples

Characteristics	BD-I (N = 24)	BD-II (N = 30)	HC (N = 45)	F or χ	df	P
Age (years), M ± SD	43.2 ± 13.7	39.5 ± 12.4	41.5 ± 13.1	0.619	2	0.54
Males, n (%)	18 (75)	19 (63.33)	25 (55.55)	2.54	2	0.281
IQ, M ± SD	103 ± 13	108 ± 9.46	110 ± 9.05	2.00	2	0.151
Duration of the illness (years), mean ± SD	17.8 ± 11.4	12.8 ± 10.5		2.67	1	0.109
Childhood-onset, n (%)	7 (29.16)	11 (36.66)		0.338	1	0.561
Current mood state:						
*Euthymia, n (%)*	18 (75)	28 (93.3)		1.48	1	0.224
*Depression, n (%)*	2 (8.3)	2 (6.7)		0.054	1	0.816
*Hypomania, n (%)*	2 (8.3)	0		2.60	1	0.107
*Mania/mixed n (%)*	2 (8.3)	0		2.60	1	0.107
Previous psychotic symptoms	17 (70.8)	1 (3.3)		27.3	1	< 0.001
Familiarity for BD n (%)	16 (66.7)	19 (63.3)		0.0650	1	0.799
Number of past episodes:						
*Depressive*						
No episodes	8	1				
Single episode	0	2				
Recurrent episodes	13	20				
*Manic*						
No episodes	1	21				
Single episode	9	0				
Recurrent episodes	11	0				
*Mixed*						
No episodes	14	21				
Single episode	5	1				
Recurrent episodes	2	0				
Time since the last episode (months), M ± SD	39.8 ± 65.1	11.1 ± 7.47		4.81	1	0.034
HAMD, M ± SD	3.9 ± 8.99	1.63 ± 2.22		1.45	1	0.236
HAMA, M ± SD	3.9 ± 8.01	1.38 ± 1.88		2.26	1	0.140
MADRS, M ± SD	5.0 ± 11.8	2.08 ± 4.17		1.28	1	0.264
YMRS, M ± SD	4.57 ± 11.2	1.04 ± 2.40		2.25	1	0.141
PANSS, M ± SD	2.95 ± 8.36	0				
GAF	61.7 ± 26.6	79.1 ± 10.1		10.2	1	0.003
Past pharmacotherapy						
*Antidepressants, n (%)*	10 (41.7)	13 (43.3)		0.015	1	0.902
*Antipsychotics, n (%)*	15 (62.5)	11 (36.7)		3.56	1	0.059
*Antiepileptics, n (%)*	7 (29.2)	4 (13.3)		2.06	1	0.151
*Benzodiazepines, n (%)*	13 (54.2)	15 (50)		0.093	1	0.761
Current pharmacotheraphy						
*Antidepressants, n (%)*	7 (29.2)	22 (73.3)		10.5	1	0.001
*Antipsychotics, n (%)*	15 (62.5)	10 (33.3)		4.56	1	0.033
*Antiepileptics, n (%)*	6 (25)	4 (13.3)		1.20	1	0.273
*Lithium, n (%)*	24 (100)	30 (100)				
Lithium treatment duration (months), M ± SD	60.2 ± 87.9	27.4 ± 40.1		3.11	1	0.084
Lithium plasma level (mmol/L), M ± SD	0.574 ± 0.191	0.521 ± 0.170		1.09	1	0.301

IQ, intelligence quotient; HAMD, Hamilton Rating Scale for Depression; HAMA, Hamilton Rating Scale for Anxiety; MADRS, Montgomery and Asberg Depression Rating Scale; YMRS, Young Mania Rating Scale; PANSS, Positive and Negative Syndrome Scale; GAF, Global Assessment of Functioning; M, mean; SD, standard deviation

### Neuropsychology

There was a main effect of group (p = 0.032) on the completion time at the TOH, with BD-I spending significantly longer time to complete the task relative to HC (p = 0.032), while no significant differences emerged between controls and BD-II (p = 0.218) and between patient subgroups (p = 0.532). There was a main effect of group on the reaction time (p = 0.004) and the rate-corrected accuracy at the FER task, with BD-I having longer reaction time and lower rate-corrected accuracy (Vandierendonck, 2017) relative to BD-II (p = 0.029, p = 0.017) and HC (p = 0.042, p = 0.017). No differences between these latter groups were found (p > 0.2) (Table 2).

**Table 2:** Neuropsychological test performance of the samples

	BD-I	BD-II	HC	F	p
TOH, completion time (msec), M ± SD	5143 ± 2086	4498 ± 2265	3683 ± 1631	3.64	0.032
FER, rate-corrected accuracy (a.u.), M ± SD	0.023 ± 0.007	0.031 ± 0.009	0.031 ± 0.010	6.440	0.004
FER, mean time (msec), M ± SD	3780 ± 1666	2895 ± 910	2989 ± 819	3.799	0.027

a.u., arbitrary units; TOH, Tower of Hanoi; FER, Facial emotion recognition task; M, mean; SD, standard deviation

### Voxel-based morphometry

**Fig. 1:**
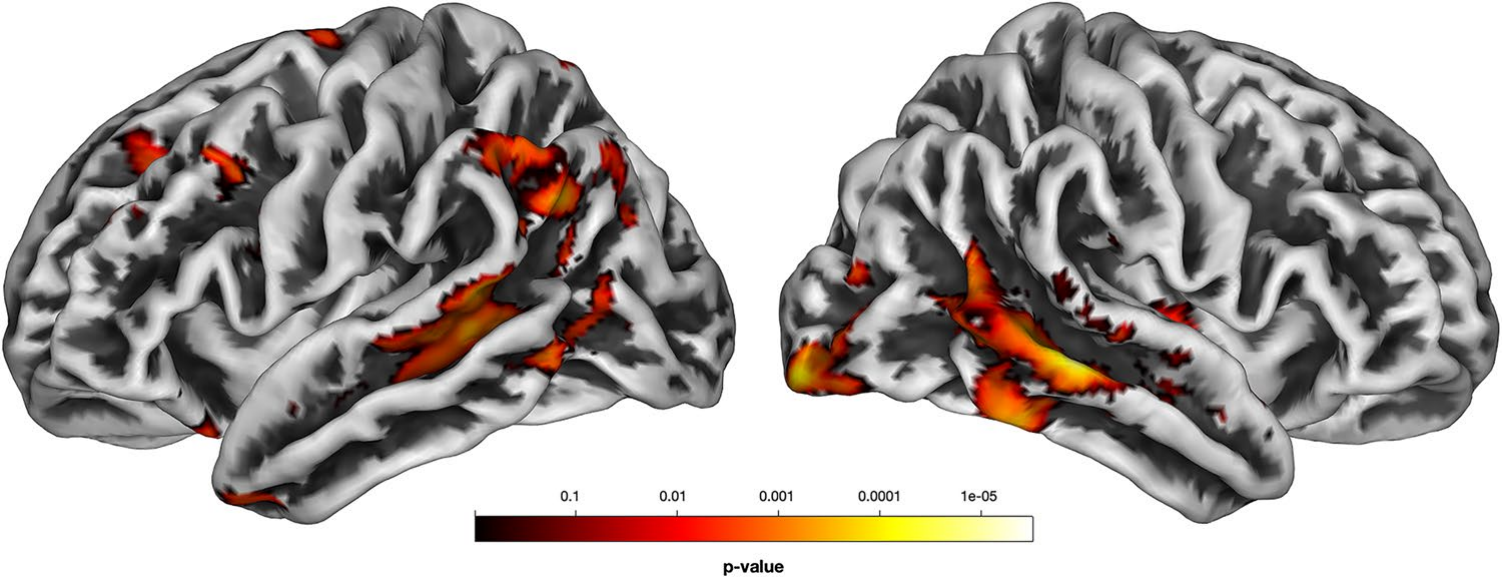
**Spatial maps of gray matter loss of patients with BD-I relative to controls.** The maps of the left and right hemisphere, respectively, are rendered on an MNI template with a voxel-wise threshold of voxel-wise p < 0.005 uncorrected for display purposes. MNI, Montreal Neurological Institute. The color bar indicates p values

Compared to HC, BD-I showed seven clusters of significant GMV reduction located bilaterally in the superior, middle, and inferior temporal gyri, in the right middle and inferior occipital gyrus, right insula, left inferior parietal lobule, and culmen (see Fig. 1, and Table 4). We did not find any GMV difference between BD-II and the other diagnostic groups.

### Source-based morphometry

#### Component selection

**Fig. 2:**
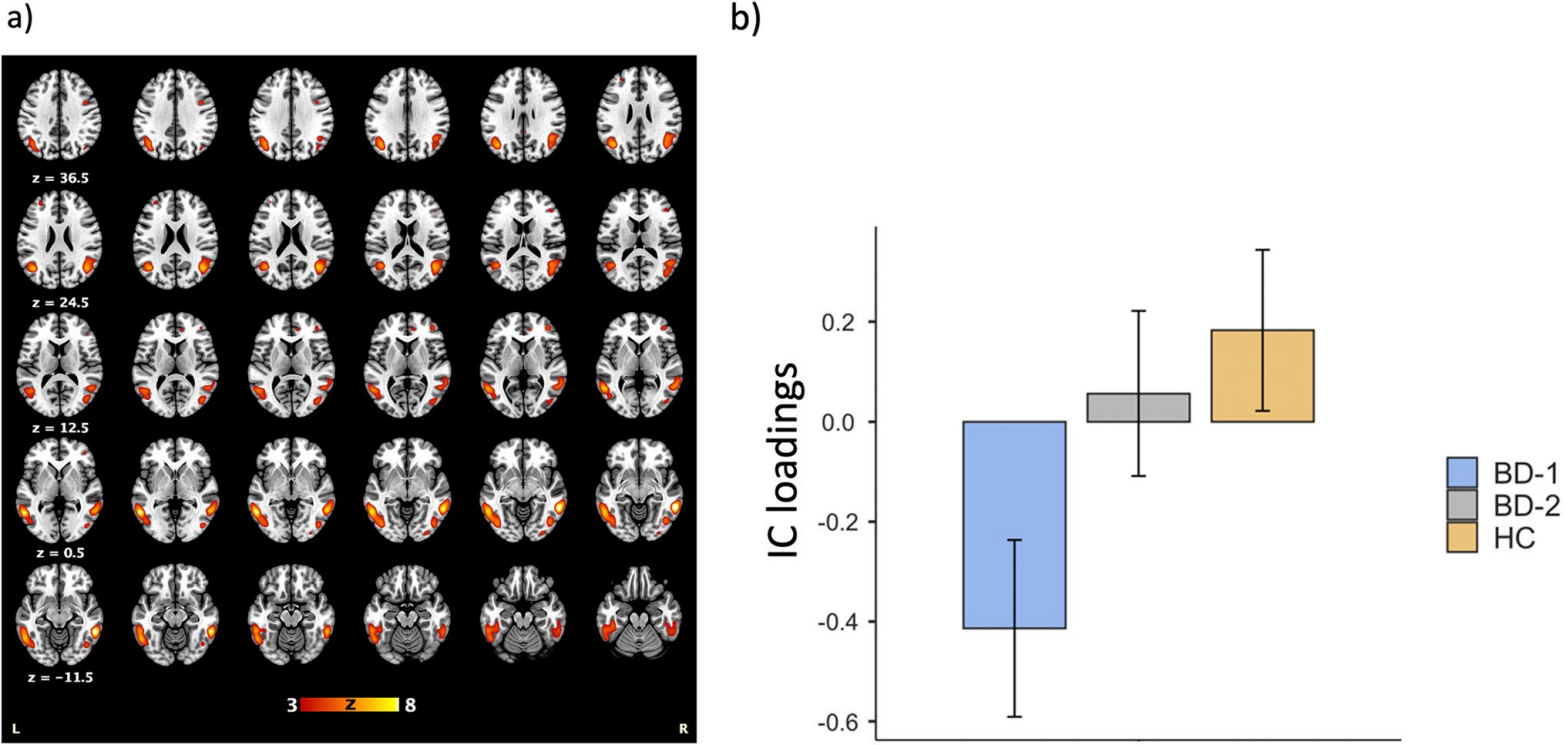
**The SBM component (IC19) involving a prefrontal-temporal-occipital network (a) exhibited significantly lower GMV covariance in BD-I compared to healthy controls, but not to BD-II (b).** Transversal 2-mm slices (axial planes, z, are indicated in the first column) are displayed on a template from the Montreal Neurological Institute. L and R indicate the left and right brain hemispheres, respectively. The color bar indicates Z-scores. The Y-axis indicates the IC loadings for the SBM component measured in arbitrary units (a.u.)

Twenty components were estimated. IC19 was correlated with GMV changes (r = 0.2) and encompassed predominantly the superior, middle, and inferior temporal gyri, precuneus, middle and inferior occipital regions, inferior parietal lobe, fusiform gyrus, inferior frontal gyrus, and anterior cingulate (see Fig. 2 and Table 3 for anatomical details and Z-scores [supplementary materials]). IC13 was also correlated with VBM changes (r = 0.15) and spanned predominantly across the temporal, precuneus, cingulate, and insular cortex.

#### Between-group analyses

IC19 loadings showed a significant effect of diagnosis (F(2,96) = 4.21, p = 0.018), and planned comparisons revealed significant differences only between HC and BD-I (p = 0.028). IC13 loadings showed a significant effect of diagnosis (F(2,96) = 3.33, p = 0.040)), and planned comparisons revealed only a trend for significance for the comparison between HC and BD-I (p = 0.067). These results were replicated when including gender as a covariate and in the euthymic subsample (see SuCite ESM.pplementary Materials).

### Brain behavior correlations

**Fig. 3:**
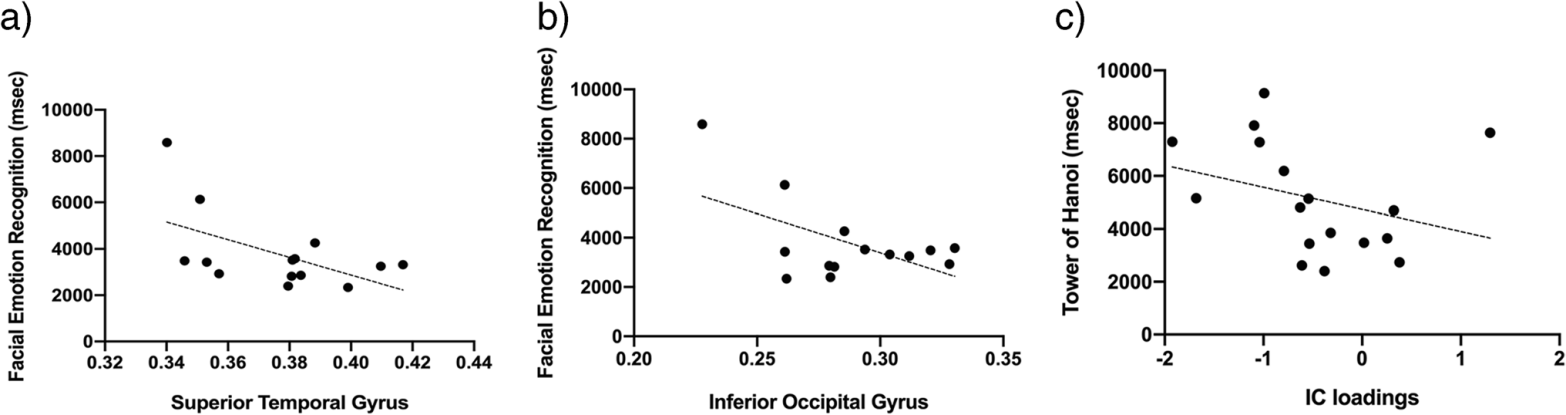
**Scatter plots of gray matter and cognition performance in patients with BD-I.** The average reaction time at the Facial emotion recognition task was inversely correlated with gray matter volume in superior temporal gyrus (a) and inferior occipital gyrus (b), respectively. The average reaction time at the Tower of Hanoi was inversely correlated with the loadings of IC19 (c)

The number of previous manic episodes was negatively correlated with the GMV loss in the right superior, middle, and inferior temporal gyri (rho = -0.463, p = 0.026), and with the IC19 loadings (rho = -0456, p = 0.029). In BD-I, FER response time was negatively correlated with GMV in the superior temporal gyrus (r = -0.545, p = 0.044; Fig. 3.a), and in the inferior occipital gyrus (r = -0.557, p = 0.039; Fig. 3.b). Moreover, the completion time of TOH in BD-I was negatively correlated with IC19 loadings (rho = -0.510, p = 0.039; Fig. 3.c). None of the exploratory correlations of the GMV and IC loadings with other cognitive and clinical variables were significant.

## Discussion

In this study, BD showed a regional reduction in GMV as well as in structural network covariance, associated with recurrence and emotional processing. First, BD-I showed decreased GMV in the insula, as well as in the temporal-occipital-parietal cortex, and the cerebellum. Second, in the brain regions showing GMV decreases, patients with BD-I had reduced structural covariance that was associated with the number of previous manic episodes, and worse executive functions, including visuospatial processing and problem-solving abilities. Third, compared to BD-II, BD-I displayed a worse performance on facial emotion recognition and showed temporal-occipital GMV loss that correlated with facial emotion recognition impairment.

Our findings of GMV reduction in the temporal lobes in BD-I are consistent with several studies (Lu et al., 2019; Wise et al., 2017). Indeed, the temporal lobe dysfunction may be of particular importance for its role in the perception and processing of environmental stimuli (Jones et al., 2009). The lower cerebellar GMV in BD-I is consistent with previous studies (Roda et al., 2015). Accumulating evidence suggests a role for the cerebellum in emotional regulation and in executive functioning, episodic memory, and sensorimotor processing (Lin et al., 2018) that are altered in BD. Although imaging cross-sectional studies on cerebellar volumetry have shown negative or controversial results (Demirgören et al., 2019; Laidi et al., 2015), a longitudinal study indicated a progressive structural decline over 4 years that is exaggerated with multiple mood episodes (Moorhead et al., 2007).

We also found lower parietal-occipital GMV (Ivleva et al., 2017). Fusiform GMV reduction has been demonstrated previously (Moorhead et al., 2007), although increases have also been reported (Adler et al., 2005). Lower right insular GMV in BD-I is in line with a previous meta-analysis that reported this area as the most atrophic (Wise et al., 2017).

These regional findings reflect distinct aspects of a complex disorder and can be integrated into a more comprehensive framework that captures the GMV network arrangement among these regions. SBM has been suggested to be more sensitive in detecting GM atrophy than other methods such as VBM (Gupta et al., 2019). SBM takes into account interrelationships among voxels to identify structural network changes, which may be undetected using VBM (Xu et al., 2009). The present SBM findings indicate a specific pattern of structural loss predominantly involving temporal-occipital regions, fusiform gyrus, inferior frontal gyrus, inferior parietal lobule, and anterior cingulate in BD-I but not in BD-II. Notably, subjects with major depressive and borderline personality disorders share comparable impairments in emotional processing and cognition with BD (Cheavens & Heiy, 2011). Similar to our findings in BD-I, previous evidence showed reduced structural network covariance with a reduced bilateral fronto-striatal network in depression and medial temporal/frontal network in borderline personality disorder, respectively (Depping et al., 2015).

We found a prefrontal-temporal-occipital network with reduced structural network covariance in BD-I, encompassing the temporal-occipital regions that span across the dorsal stream of visual processing. Distinct streams of processing originating from primary visual areas reach the inferior temporal (ventral) and posterior parietal areas (dorsal) (Ray et al., 2020). The ventral stream has been implicated in object identification (“what”) along with its internal representation (“perception”). Conversely, the perception of spatial information (“where”) and guiding visuomotor 'actions' (Ray et al., 2020) are processed in the occipitoparietal or dorsal stream (Haxby et al., 1991). Notably, we found an association between reduced executive functions involving visuospatial performance and problem-solving abilities, as demonstrated by a worse performance of TOH, and the structural network covariance loss in BD-I, but not in BD-II and HCs.

Occipital-temporal regions are also implicated in face processing. The face-selective network is composed of a dorsal pathway from the occipital face area to the superior temporal sulcus (STS) and a ventral pathway from the occipital face area to the fusiform. The fusiform, and the posterior-STS constitute the core system of face processing (Haxby & Ida Gobbini, 2007). The inferior occipital cortex is responsible for the early stages of face processing and sends its output to both the fusiform, involved in the representation of invariant aspects of the face (e.g., face identity), and the posterior-STS involved in the representation of changeable aspects (e.g., facial expression, eye-gaze, etc.; Bernstein & Yovel, 2015). The ventral visual stream is strongly influenced by emotional stimuli (Vuilleumier, 2005) and displays both functional and anatomical connectivity with the amygdala (Amaral et al., 2003). Therefore, in BD-I, GMV loss in the STS and in the inferior occipital gyrus may be associated with impaired emotional processing as suggested also by the association with FER performance.

An inverse correlation between the number of manic episodes and the reduced structural network covariance was found. This finding is consistent with the correlation between the main cluster of GMV loss involving the right temporal regions and the number of manic episodes that emerged in our BD-I sample using VBM. Previous literature has demonstrated a significant correlation between the number of manic episodes and prefrontal GMV reduction (Ekman et al., 2010). However, the involvement of right temporal GMV reduction is in line with a recent study on lesional mania that showed an overrepresentation of right occipital, and parietal lesions, with the fusiform gyrus more frequently involved (Barahona-Corrêa et al., 2020). Such a wide distribution of GM areas argues for a circuit-based impact of the lesions associated with secondary mania similar with the prefrontal-temporal-occipital network in BD-I.

The study suffers from some limitations. First, although most of the patients were in the euthymic state, we included patients with depression at the time of evaluation. Although brain-behavior correlations may be partly affected, the current mood state did not show effects on brain structure (Foland-Ross et al., 2012). Second, the cross-sectional study design limits our ability to make inferences about a causal relationship between abnormal structural connectivity and behavior. Third, the study did not include drug-free patients. Due to the disorder severity, patients mantain pharmacological treatments even in euthymia that may affect neuroimaging measures. Lithium exposure may increase GMV specifically in the limbic system (Sani et al., 2018). However, in our study, lithium exposure was not different between BD-subtypes. Moreover, it is known that the use of antipsychotics may reduce GMV and impair neuropsychological performance (Centorrino et al., 2005). Nonetheless, no significant correlations between antipsychotics doses and IC-loadings and with neuropsychological scores emerged in our sample.

Overall, our work supports the hypothesis that BD may be associated with impaired visual processing (Reavis et al., 2020) and provides evidence of structural abnormalities of the visual system in BD-I similar to other disorders of the psychosis spectrum (Reavis et al., 2020). Furthermore, the association between structural changes and impairments in cognitive and social-cognitive domains may contribute to functional deficits and clinical outcomes in BD-I. Lastly, it has been suggested that emotional regulation and cognitive function reciprocally interact (Lima et al., 2018), so that impaired cognition may affect negatively emotional processing, as well as heightened emotional status, can impair cognitive functions in BD (Lima et al., 2018). Consistent with this, the present study suggests that altered structural covariance of the temporo-occipital network in BD-I may be involved in the interplay between cognitive control and emotional processing, whereby altered structure results in dysfunctional integrative processing of mood and cognition.

## Conclusions

This study provides evidence for a pattern of regions of GM covariance loss in BD-I. The abnormal prefrontal-temporal-occipital network is associated with disease severity and may reflect a neural signature associated with an impairment in executive abilities, as well as emotion processing, in BD-I. Future studies with a longitudinal design could help to address inferences about the causal directions of the relationships emerging between the pattern of abnormal structural network covariance and cognitive and clinical data in BD.

## Supplementary Information

Below is the link to the electronic supplementary material.

Supplementary file1 (DOCX 24 kb)

Supplementary file2 (PNG 4892 kb)

Supplementary file3 (PNG 5426 kb)

Supplementary file4 (PNG 251 kb)

## Data Availability

The data that support the findings of this study are available from the corresponding author upon reasonable request.

## References

[CR1] Abé C, Ekman C-J, Sellgren C, Petrovic P, Ingvar M, Landén M (2016). Cortical thickness, volume and surface area in patients with bipolar disorder types I and II. Journal of Psychiatry & Neuroscience : JPN.

[CR2] Adler CM, Levine AD, DelBello MP, Strakowski SM (2005). Changes in gray matter volume in patients with bipolar disorder. Biological Psychiatry.

[CR3] Amaral DG, Behniea H, Kelly JL (2003). Topographic organization of projections from the amygdala to the visual cortex in the macaque monkey. Neuroscience.

[CR4] Baldessarini RJ, Tondo L, Baethge CJ, Lepri B, Bratti IM (2007). Effects of treatment latency on response to maintenance treatment in manic-depressive disorders. Bipolar Disorders.

[CR5] Barahona-Corrêa, J. B., Cotovio, G., Costa, R. M., Ribeiro, R., Velosa, A., Silva, V. C. e., Sperber, C., Karnath, H.-O., Senova, S., & Oliveira-Maia, A. J. (2020). Right-sided brain lesions predominate among patients with lesional mania: Evidence from a systematic review and pooled lesion analysis. *Translational Psychiatry*, *10*. 10.1038/s41398-020-0811-0 10.1038/s41398-020-0811-0 PMC7217919 32398699

[CR6] Bernstein M, Yovel G (2015). Two neural pathways of face processing: A critical evaluation of current models. Neuroscience and Biobehavioral Reviews.

[CR7] Bora E, Fornito A, Yücel M, Pantelis C (2012). The effects of gender on grey matter abnormalities in major psychoses: A comparative voxelwise meta-analysis of schizophrenia and bipolar disorder. Psychological Medicine.

[CR8] Bora E, Özerdem A (2017). Meta-analysis of longitudinal studies of cognition in bipolar disorder: Comparison with healthy controls and schizophrenia. Psychological Medicine.

[CR9] Bora E (2018). Neurocognitive features in clinical subgroups of bipolar disorder: A meta-analysis. Journal of Affective Disorders.

[CR10] Cheavens JS, Heiy J (2011). The differential roles of affect and avoidance in major depressive and borderline personality disorder symptoms. Journal of Social and Clinical Psychology.

[CR11] Centorrino F, Fogarty KV, Cimbolli P, Salvatore P, Thompson TA, Sani G, Cincotta SL, Baldessarini RJ (2005). Aripiprazole: Initial clinical experience with 142 hospitalized psychiatric patients. Journal of Psychiatric Practice.

[CR12] Demirgören BS, Özbek A, Karabekir NG, Ay B, Turan S, Yonguç GN, Karabekir S, Polat Aİ, Hız AS, Kıdak ÖG (2019). Cerebellar volumes in early-onset bipolar disorder: A pilot study of a stereological measurement technique. Psychiatry and Clinical Psychopharmacology.

[CR13] Depping MS, Wolf ND, Vasic N, Sambataro F, Thomann PA, Wolf RC (2015). Common and distinct structural network abnormalities in major depressive disorder and borderline personality disorder. Progress in Neuro-Psychopharmacology and Biological Psychiatry.

[CR14] Ekman CJ, Lind J, Rydén E, Ingvar M, Landén M (2010). Manic episodes are associated with grey matter volume reduction—A voxel-based morphometry brain analysis. Acta Psychiatrica Scandinavica.

[CR15] Foland-Ross LC, Brooks JO, Mintz J, Bartzokis G, Townsend J, Thompson PM, Altshuler LL (2012). Mood-state effects on amygdala volume in bipolar disorder. Journal of Affective Disorders.

[CR16] Gupta CN, Turner JA, Calhoun VD (2019). Source-based morphometry: A decade of covarying structural brain patterns. Brain Structure & Function.

[CR17] Hamilton M (1959). The assessment of anxiety states by rating. The British Journal of Medical Psychology.

[CR18] Hamilton M (1960). A rating scale for depression. Journal of Neurology, Neurosurgery, and Psychiatry.

[CR19] Haxby JV, Grady CL, Horwitz B, Ungerleider LG, Mishkin M, Carson RE, Herscovitch P, Schapiro MB, Rapoport SI (1991). Dissociation of object and spatial visual processing pathways in human extrastriate cortex. Proceedings of the National Academy of Sciences of the United States of America.

[CR20] Haxby JV, Ida Gobbini M (2007). The perception of emotion and social cues in faces. Neuropsychologia.

[CR21] Hibar, D. P., Westlye, L. T., Doan, N. T., Jahanshad, N., Cheung, J. W., Ching, C. R. K., Versace, A., Bilderbeck, A. C., Uhlmann, A., Mwangi, B., Krämer, B., Overs, B., Hartberg, C. B., Abé, C., Dima, D., Grotegerd, D., Sprooten, E., Bøen, E., Jimenez, E., … Andreassen, O. A. (2018). Cortical abnormalities in bipolar disorder: An MRI analysis of 6503 individuals from the ENIGMA Bipolar Disorder Working Group. *Molecular Psychiatry*, *23*(4), 932–942. 10.1038/mp.2017.73 10.1038/mp.2017.73 PMC5668195 28461699

[CR22] Hirschfeld RMA, Calabrese JR, Weissman MM, Reed M, Davies MA, Frye MA, Keck PE, Lewis L, McElroy SL, McNulty JP, Wagner KD (2003). Screening for bipolar disorder in the community. The Journal of Clinical Psychiatry.

[CR23] Işık Ulusoy S, Gülseren ŞA, Özkan N, Bilen C (2020). Facial emotion recognition deficits in patients with bipolar disorder and their healthy parents. General Hospital Psychiatry.

[CR24] Ivleva EI, Clementz BA, Dutcher AM, Arnold SJM, Jeon-Slaughter H, Aslan S, Witte B, Poudyal G, Lu H, Meda SA, Pearlson GD, Sweeney JA, Keshavan MS, Tamminga CA (2017). Brain Structure Biomarkers in the Psychosis Biotypes: Findings From the Bipolar-Schizophrenia Network for Intermediate Phenotypes. Biological Psychiatry.

[CR25] Jones LD, Payne ME, Messer DF, Beyer JL, MacFall JR, Krishnan KRR, Taylor WD (2009). Temporal lobe volume in bipolar disorder: Relationship with diagnosis and antipsychotic medication use. Journal of Affective Disorders.

[CR26] Kay SR, Fiszbein A, Opler LA (1987). The positive and negative syndrome scale (PANSS) for schizophrenia. Schizophrenia Bulletin.

[CR27] Keck PE, Kessler RC, Ross R (2008). Clinical and economic effects of unrecognized or inadequately treated bipolar disorder. Journal of Psychiatric Practice.

[CR28] Laidi C, d’Albis M-A, Wessa M, Linke J, Phillips ML, Delavest M, Bellivier F, Versace A, Almeida J, Sarrazin S, Poupon C, Le Dudal K, Daban C, Hamdani N, Leboyer M, Houenou J (2015). Cerebellar volume in schizophrenia and bipolar I disorder with and without psychotic features. Acta Psychiatrica Scandinavica.

[CR29] Lima IMM, Peckham AD, Johnson SL (2018). Cognitive deficits in bipolar disorders: Implications for emotion. Clinical Psychology Review.

[CR30] Lin K, Shao R, Lu R, Chen K, Lu W, Li T, Kong J, So K-F, Xu G (2018). Resting-state fMRI signals in offspring of parents with bipolar disorder at the high-risk and ultra-high-risk stages and their relations with cognitive function. Journal of Psychiatric Research.

[CR31] Lu X, Zhong Y, Ma Z, Wu Y, Fox PT, Zhang N, Wang C (2019). Structural imaging biomarkers for bipolar disorder: Meta-analyses of whole-brain voxel-based morphometry studies. Depression and Anxiety.

[CR32] Maller JJ, Thaveenthiran P, Thomson RH, McQueen S, Fitzgerald PB (2014). Volumetric, cortical thickness and white matter integrity alterations in bipolar disorder type I and II. Journal of Affective Disorders.

[CR33] Merikangas KR, Akiskal HS, Angst J, Greenberg PE, Hirschfeld RMA, Petukhova M, Kessler RC (2007). Lifetime and 12-month prevalence of bipolar spectrum disorder in the National Comorbidity Survey replication. Archives of General Psychiatry.

[CR34] Montgomery, S. A., & Asberg, M. (1979). A new depression scale designed to be sensitive to change. The British Journal of Psychiatry: *The Journal of Mental Science,* 134, 382–389. 10.1192/bjp.134.4.382 10.1192/bjp.134.4.382 444788

[CR35] Moorhead TWJ, McKirdy J, Sussmann JED, Hall J, Lawrie SM, Johnstone EC, McIntosh AM (2007). Progressive gray matter loss in patients with bipolar disorder. Biological Psychiatry.

[CR36] Ray D, Hajare N, Roy D, Banerjee A (2020). Large-scale Functional Integration, Rather than Functional Dissociation along Dorsal and Ventral Streams, Underlies Visual Perception and Action. Journal of Cognitive Neuroscience.

[CR37] Reavis, E. A., Lee, J., Altshuler, L. L., Cohen, M. S., Engel, S. A., Glahn, D. C., Jimenez, A. M., Narr, K. L., Nuechterlein, K. H., Riedel, P., Wynn, J. K., & Green, M. F. (2020). Structural and Functional Connectivity of Visual Cortex in Schizophrenia and Bipolar Disorder: A Graph-Theoretic Analysis. *Schizophrenia Bulletin Open*, *1*(1). 10.1093/schizbullopen/sgaa056 10.1093/schizbullopen/sgaa056 PMC7712743 33313506

[CR38] Roda Â, Chendo I, Kunz M (2015). Biomarkers and staging of bipolar disorder: A systematic review. Trends in Psychiatry and Psychotherapy.

[CR39] Sani G, Simonetti A, Janiri D, Banaj N, Ambrosi E, De Rossi P, Ciullo V, Arciniegas DB, Piras F, Spalletta G (2018). Association between duration of lithium exposure and hippocampus/amygdala volumes in type I bipolar disorder. Journal of Affective Disorders.

[CR40] Srivastava C, Bhardwaj A, Sharma M, Kumar S (2019). Cognitive Deficits in Euthymic Patients With Bipolar Disorder: State or Trait Marker?. The Journal of Nervous and Mental Disease.

[CR41] Vandierendonck A (2017). A comparison of methods to combine speed and accuracy measures of performance: A rejoinder on the binning procedure. Behavior Research Methods.

[CR42] Vuilleumier P (2005). How brains beware: Neural mechanisms of emotional attention. Trends in Cognitive Sciences.

[CR43] Wang X, Luo Q, Tian F, Cheng B, Qiu L, Wang S, He M, Wang H, Duan M, Jia Z (2019). Brain grey-matter volume alteration in adult patients with bipolar disorder under different conditions: A voxel-based meta-analysis. Journal of Psychiatry & Neuroscience: JPN.

[CR44] Wise T, Radua J, Via E, Cardoner N, Abe O, Adams TM, Amico F, Cheng Y, Cole JH, de Azevedo Marques Périco, C., Dickstein, D. P., Farrow, T. F. D., Frodl, T., Wagner, G., Gotlib, I. H., Gruber, O., Ham, B. J., Job, D. E., Kempton, M. J., … Arnone, D.  (2017). Common and distinct patterns of grey-matter volume alteration in major depression and bipolar disorder: Evidence from voxel-based meta-analysis. Molecular Psychiatry.

[CR45] Xu L, Groth KM, Pearlson G, Schretlen DJ, Calhoun VD (2009). Source-based morphometry: The use of independent component analysis to identify gray matter differences with application to schizophrenia. Human Brain Mapping.

[CR46] Young RC, Biggs JT, Ziegler VE, Meyer DA (1978). A rating scale for mania: Reliability, validity and sensitivity. The British Journal of Psychiatry: The Journal of Mental Science.

