# Supplementary material for: Gray matter volume covariance networks are associated with altered emotional processing in bipolar disorder: a source-based morphometry study
Source: Brain Imaging Behav. 2021 Sep 21;16(2):738–47. doi: 10.1007/s11682-021-00541-5 (PMC9010334; doi:10.1007/s11682-021-00541-5)
Supplement: Supplementary file 1 — Supplementary file1 (DOCX 24 kb) [file 11682_2021_541_MOESM1_ESM.docx]

**Supplementary Materials**

**Gray matter volume covariance networks are associated with altered emotional processing in bipolar disorder: a source-based morphometry study**

Alessandro Miola^1^, Nicolò Trevisan^1^, Arcangelo Merola^1^, Francesco Folena Comini^1^, Daniele Olivo^1^, Matteo Minerva^1^, Silvia Valeggia^1^, Tommaso Toffanin^1^, Angela Favaro^1,2^, Renzo Manara^1,2^, Fabio Sambataro^1,2^

^1^ Department of Neuroscience (DNS), University of Padova, Italy

^2^ Padova Neuroscience Center, University of Padova, Italy

Corresponding author:

Fabio Sambataro

Department of Neuroscience (DNS), University of Padova, Italy

Via Giustiniani 5, Padova

[Tel:+390498211980](about:blank)

e-mail: fabio.sambataro@unipd.it

**METHODS**

**Participants**

Fifty-seven patients with BD and 45 age, gender, handedness, and IQ matched healthy controls (HC) participated in this study. Edinburgh handedness inventory and Raven Matrices for the Intelligence Quotient were used to assess handedness and IQ, respectively. Patients were recruited from the Psychiatric Ward and Mood disorders inpatient service of the University Hospital, between January 2020 and October 2020. Patients were diagnosed with BD using the Structured Clinical Interview for DSM-5-Patient Edition (SCID-5) and had stable drug treatment for at least 1 month. Exclusion criteria for all participants included age >65 or <18 years, history of alcohol or drug abuse in the six months before the assessment, lifetime drug dependence, traumatic head injury with loss of consciousness, past or present major medical illness, neurological disorders, and mental retardation. Additionally, controls were excluded if they had a history or current diagnosis of psychiatric disorders or drug treatment (except birth control pills in young women). Two patients were excluded for the presence of vascular-like lesions, and one patient experienced a panic attack and could not complete the scan. The final sample included 24 BD-I and 30 BD-II.

This study was approved by the local Ethics Committee and was carried out in accordance with the guidelines of the Declaration of Helsinki of 1975. All participants gave their written informed consent to participate in the study after they had received a complete explanation of the procedures.

Data were preprocessed using the Computational Anatomy Toolbox for SPM (CAT12, http:// [www.neuro.uni-jena.de/cat/](http://www.neuro.uni-jena.de/cat/)), a toolbox running within the Statistical Parametric Mapping analysis package (SPM12, <http://www.fil.ion.ucl.ac.uk/spm/software/spm12/>). After the initial data quality check (to avoid critical artifacts such as head-motion, ghosting, and stripes that could potentially affect the results), each participant's T1 image was reoriented to the ACPC, and then was spatially normalized and segmented into gray and white matter and cerebrospinal fluid based on maximum a posteriori estimation. After data preprocessing, modulated normalized gray and white matter volumes were smoothed using an 8-mm Full-Width Half Maximum (FWHM) Gaussian kernel. We applied a 0.2 absolute masking threshold. First, a voxel-wise general linear model (GLM) with total intracranial volume and age as covariates was used to compare GMV among the three diagnostic groups (BD-I, BD-II, and controls) using pairwise univariate linear contrasts. Permutation-based nonparametric testing using the threshold-free cluster enhancement (TFCE) method (TFCE toolbox, http://dbm.neuro.uni-jena.de/tfce) with 5,000 permutations was applied to the t-stats maps to family-wise error (FWE) correct for multiple comparisons at the cluster-level with a threshold of alpha=0.05.

**Source-based morphometry**

Methodological details on SBM can be found elsewhere (Xu et al. 2009). Briefly, a spatial independent component analysis (ICA) was computed on all subjects’ preprocessed GMV images with the Infomax algorithm using the “Group ICA for fMRI Toolbox” GIFT; https://trendscenter.org/software/gift/). The ‘minimum description length’ criteria were used to estimate the number of components. To increase the stability of the estimated components, the ICASSO algorithm was used and the ICA estimation was repeated 50 times with bootstrapping and permutation. Using ICA, every GMV image was converted into a one-dimensional vector: The output of SBM is a numerical coefficient matrix composed of rows (participants) and columns (extracted components). All GMV images were arrayed into one 99-row subject-by-image matrix. This matrix was then decomposed into a source matrix, representing the relationship between components and brain voxels (i.e., the spatial maps of the GMV components), and the mixing matrix which indicates the relationship between participants and components. Subsequent between-group comparisons were performed using mixing matrix indices, i.e. loading parameters representing each individual’s contribution to the GMV components of the total sample. Components that showed reliability, as indicated by an ICASSO coefficient of stability >0.8, and were spatially correlated with at least a small effect size with clusters showing GMV changes in patients with BD (Pearson’s correlation r>0.1) were retained for group analyses. First, we calculated component-related differences in GMV between BD-I, BD-II, and controls using ANCOVAs with age and TIV as covariates in the columns of the mixing matrices of the selected components, followed by Tukey’s post-hoc test (p<0.05). For the visualization of the GMV component, the source matrix was reshaped to a three-dimensional image (i.e., the same dimension as the input images), scaled to unit standard deviations (Z maps), and thresholded at Z>3.5. Maps of components exhibiting significant differences between the groups were then overlaid onto a Montreal Neurological Institute (MNI) normalized anatomical template. Anatomical labels and stereotaxic coordinates of clusters above a threshold of Z = 3.5 were obtained using the Talairach Atlas.

**Additional analyses**

We performed a further analysis of the IC19 loadings in the BD-I vs. HC contrast using ANCOVA including gender as a covariate, together with age and TIV, to exclude the possible confounding role of the gender variable. Compared to HCs, BD-I still showed significantly reduced IC19 loadings (p=0.041).

To exclude the possible role of mood state at the time of the scan, we performed the ANCOVA only in euthymic patients with BD-I. Compared to HC, patients with BD-I showed a significant reduction in IC19 loadings (p=0.017).

**TABLE 3.** Independent components of GMV (IC) showing differences between samples.

| **Brain region** | **Brodmann area** | **L**  **Z-score (x,y,z coordinates )** | **R**  **Z-score (x,y,z coordinates** | **Volume (cc) L/R** |
| --- | --- | --- | --- | --- |
| ***IC19*** | | | | |
| Middle Temporal Gyrus | 20, 21, 22, 37, 39 | 8.6 (-56, -52, 1) | 10.0 (58, -44, -7) | 6.7/9.2 |
| Inferior Temporal Gyrus | 19, 20 | 7.0 (-55, -54, -6) | 3.6 (56, -31, -17) | 3.1/0.5 |
| Superior Temporal Gyrus | 22, 39 | 5.8 (-42, -57, 21) | 5.2 (52, -53, 15) | 2.0/2.6 |
| Angular Gyrus | 39 | 5.8 (-43, -60, 33) | - | 1.5/- |
| Sub-Gyral | 18, 19 | 5.7 (-42, -71, -1) | 4.3 (31, -88, -2) | 1.0/1.0 |
| Middle Occipital Gyrus | 19 | 5.6 (-52, -61, -6) | 4.5 (43, -76, 1) | 1.0/0.5 |
| Supramarginal Gyrus | 40 | 4.1 (-46, -52, 34) | 5.5 (46, -53, 27) | 0.5/1.0 |
| Inferior Occipital Gyrus | 17 | - | 5.4 (43, -69, -3) | -/0.5 |
| Fusiform Gyrus | 20 | 4.8 (-43, -36, -17) | 4.2 (52, -45, -17) | 2.0/1.0 |
| Inferior Frontal Gyrus | 44 | - | 4.7 (40, 45, 2) | -/1.5 |
| Precuneus | 19 | 4.1 (-39, -69, 39) | - | 1.0/- |
| Inferior Parietal Lobule | 40 | - | 3.7 (46, -45, 24) | -/0.5 |
| Anterior Cingulate | 32 | - | 3.7 (10, 42, 5) | -/0.5 |

For each hemisphere (left = L, right = R) the peak Z-value and stereotaxic Montreal Neurological Institute (MNI) coordinates (x, y, z) are provided. The volume of the voxels in each area is provided in cubic centimeters (cc).

**TABLE 4.** Brain regions showing gray matter volume loss in patients with BD-I and controls.

| **Peak Brain region** | **BA** | **Cluster regional extent** | **Cluster size** | **Peak Coordinates (x,y,z)** |
| --- | --- | --- | --- | --- |
| Middle temporal gyrus | 21 | Right middle, superior, inferior temporal gyrus, fusiform gyrus, and subgyral. | 4213 | 65, -33, 1 |
| Superior temporal gyrus | 22 | Left middle, superior temporal gyrus, and sub-gyral. | 1268 | -48, -40, 1 |
| Superior temporal gyrus |  | Right superior temporal gyrus, sub-gyral, and right insula | 1102 | 40, -9, -11 |
| Inferior occipital gyrus | 19 | Right inferior, middle occipital gyrus. | 800 | 33, -90, -5 |
| Supramarginal gyrus | 40 | Left inferior parietal lobule, supramarginal gyrus, and angular gyrus. | 500 | -43, -58, -5 |
| Culmen |  | Right cerebellum anterior lobe, culmen. | 296 | 22, -49, 22 |
| Middle temporal gyrus | 21 | Right middle temporal gyrus, sub-gyral. | 138 | 49, -14, 23 |

Peak stereotaxic coordinates are reported in Montreal Neurological Institute (MNI) system. BA, Brodmann Area
