# Supplementary figures and images for: Gray matter volume covariance networks are associated with altered emotional processing in bipolar disorder: a source-based morphometry study
Source: Brain Imaging Behav. 2021 Sep 21;16(2):738–47. doi: 10.1007/s11682-021-00541-5 (PMC9010334; doi:10.1007/s11682-021-00541-5)

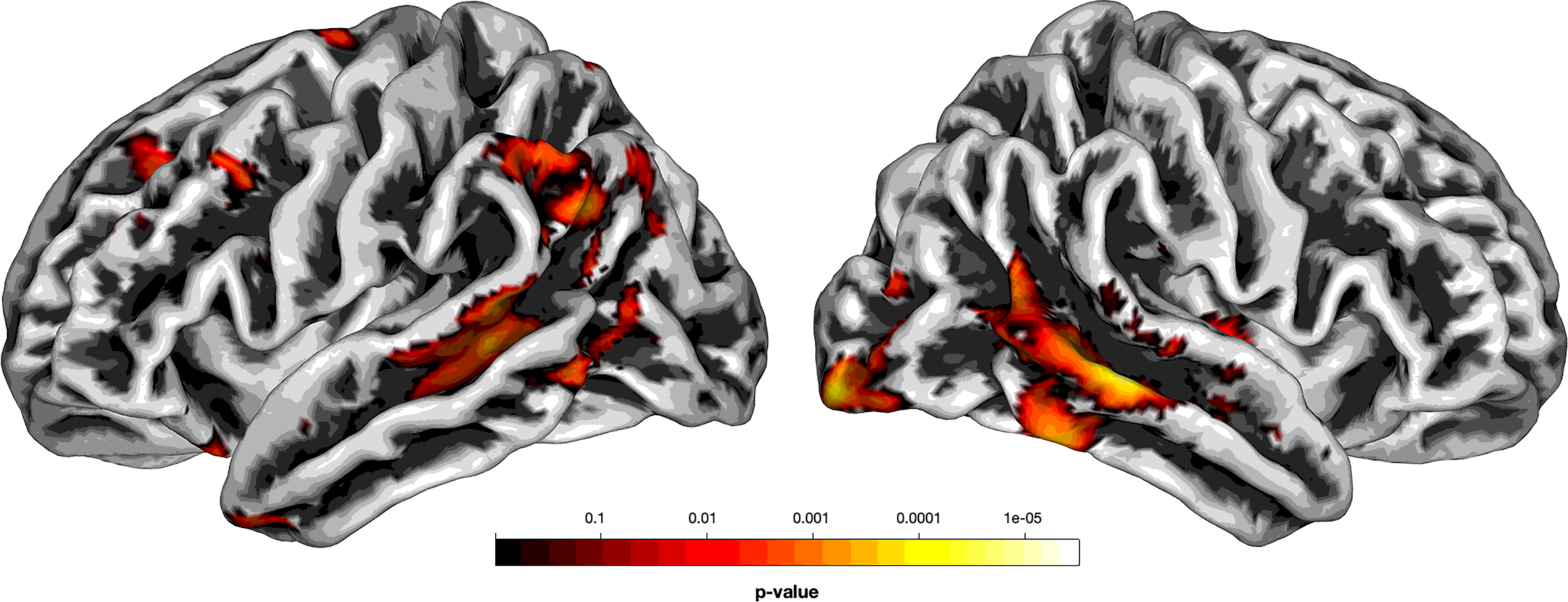

Supplement: Supplementary file 2 — Supplementary file2 (PNG 4892 kb) [file 11682_2021_541_MOESM2_ESM.png]

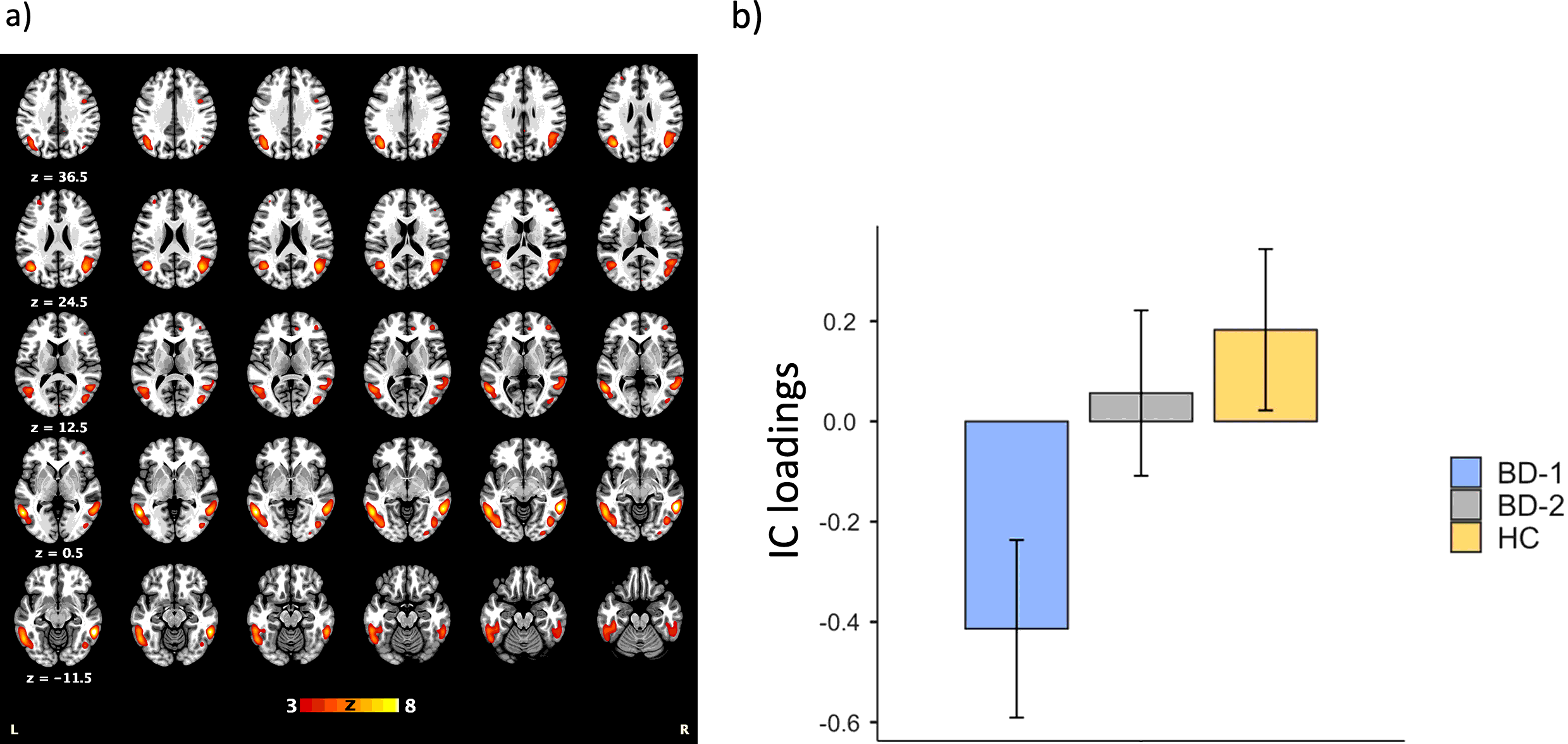

Supplement: Supplementary file 3 — Supplementary file3 (PNG 5426 kb) [file 11682_2021_541_MOESM3_ESM.png]

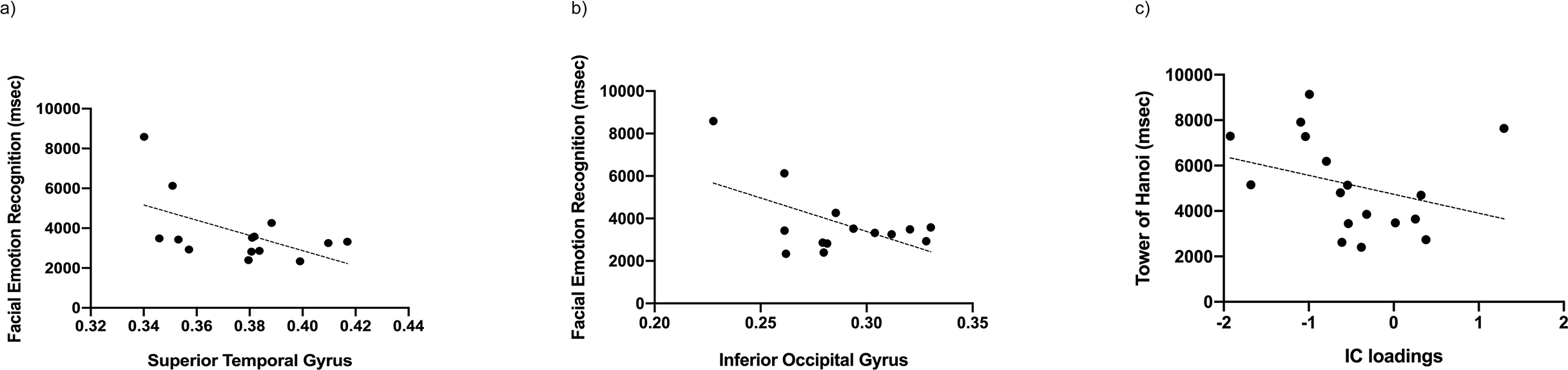

Supplement: Supplementary file 4 — Supplementary file4 (PNG 251 kb) [file 11682_2021_541_MOESM4_ESM.png]
